# Selection of Tubular and Endoscopic Transforaminal Disc Procedures Based on Disc Size, Location, and Characteristics

**DOI:** 10.7759/cureus.2091

**Published:** 2018-01-20

**Authors:** Ovidiu Palea, Michelle Granville, Robert E Jacobson

**Affiliations:** 1 Anesthesiology and Pain Management, Provita Hospital; 2 Miami Neurosurgical Center, University of Miami Hospital

**Keywords:** endoscopic disectomy, minimally invasive discectomy, percutaneous interbody fusion, extruded disc, foraminal disc, percutaneous discectomy, herniated disc, recurrent disc herniation

## Abstract

The clinical effectiveness of percutaneous and transforaminal endoscopic discectomy procedures has been evaluated by the system used or compared to open laminectomy or micro-discectomy but are not evaluated based on the location and characteristics of the abnormal disc. This review proposes that outcomes are primarily related to disc size, biomechanics, location, and associated segmental fibrotic and bone changes as well as the surgeon's skill in using various systems rather than the specific system used. In these cases, the surgeon needs to decide if the goal of the procedure is simply internal decompression of an abnormal but contained herniated disc or release of the entrapped nerve root by a large contained disc, extruded and migrated disc fragment, or coexistent foraminal stenosis. Percutaneous and tubular transforaminal procedures are quite different, technically ranging from simple discectomy aspirating probes to larger endoscopic systems, providing the capability to remove large extruded free disc fragments, with or without foraminotomy. Recently, the ability to perform interbody fusion has been added to the range of procedures able to be performed endoscopically. At the same time, biologic solutions to disc degeneration are rapidly evolving and may have a place in combination with these procedures. This article reviews the interrelationship between clinical signs and symptoms, radiologic findings, and the biochemistry and biomechanics of the affected disc segment. Understanding the role played by all these factors enables the surgeon to evaluate both the disc and surrounding bone structures pre-operatively to determine if the clinical signs and symptoms are related to enlargement and displacement of a contained disc or compression or impingement of the nerve root. Based on this, the surgeon can choose different surgical systems, allowing simple decompression of a contained disc, possibly adding biologics, with a 'small' system, while a large herniated disc, or extruded fragment, causing root impingement, would require a ‘larger’ system that provides direct endoscopic visualization within the epidural space, foraminal decompression with drills, and direct surgical manipulation and freeing of the nerve root. By choosing the surgical system based on characteristics such as disc size, location, and associated inflammatory and fibrotic changes, the effectiveness of minimally invasive procedures will be more consistent and improve as the surgeon's diagnostic and operative skills improve.

## Introduction and background

The literature regarding percutaneous and transforaminal endoscopic discectomy has focused on the effectiveness of specific systems rather than how the actual procedure performed relates to the underlying disc pathology [1]. Disc pathology is a continuum of disease where one form of disc pathology evolves into another, often with long asymptomatic periods, and where there can also be a mixture of secondary facet and bone changes evolving along with the original disc pathology. Each stage of disc disease has unique characteristics and varies in its correlation with discogenic pain, radicular pain, and development of adjacent tissue irritation and degeneration [2]. The primary pathology in intervertebral discogenic disease (IDD) begins with fissuring of the annulus, though some nuclear degeneration also occurs. Over time, changing characteristics of the abnormal disc plus disc space narrowing alters the biomechanics of the spinal segment. At the same time, inflammation and degeneration of nearby tissues including the facet joints can cause or aggravate the symptoms of the patient. Currently, many studies are targeting these early stages of disc degeneration with biologic treatments in an attempt to arrest or even reverse disease progression [2-3]. Comparing the effectiveness of different procedures is difficult since patients not only have different radiologic findings but clinically vary tremendously [4-6].  Before examining the reasons for adopting different surgical systems based on the underlying radiologic segmental spinal pathology, it is critical to understand the interrelationship between clinical symptomatology, radiologic findings, spinal biomechanics, and what is known regarding the physiologic, biochemical, and inflammatory response within the abnormal disc and surrounding tissues. This helps guide the surgeon using minimally invasive and transforaminal surgical procedures to decide whether the surgical approach should be primarily intra-discal, only targeting the nucleus and disc annulus, or more epidural and transforaminal, targeting the neural foramina and any compression within the epidural space from large disc fragments besides the disc space [1-2].

## Review

The key to selecting the proper surgical procedure for symptomatic lumbar spine abnormalities has always been accurate diagnosis and understanding of the capabilities and limits of any given surgical technique in combination with the skill of the surgeon. This applies whether it is open surgery or minimally invasive and endoscopic procedures. The role of spinal biomechanics is also critical since understanding the cause of symptoms, and the effect of the surgery on spinal motion, and abnormal physiology and motion of the disc and spinal segment helps to determine the best surgical approach to the abnormal disc as well as the area causing nerve root compression. The major advantage of all minimal spinal lumbar procedures is to access and remove abnormal pathology while preserving the anatomy and function of the muscles, ligaments, and bone structures, thus providing faster patient recovery, better lumbar support, and preserving as much normal spinal motion as possible. As minimally invasive tubular and endoscopic procedures have technically advanced and become commonplace, it is also critical to understand the limits of each procedure and the fact that all preoperative decisions and surgical planning is based on detailed computerized tomography (CT) and magnetic resonance imaging (MRI) and relating these radiologic findings to the patients' signs and symptoms [7]. The surgeon is using a very limited approach to access specific pathology, usually only at one level and one side of the patient, so it is critical the diagnostic testing used is properly correlated with the various findings and stages of disc degeneration [8]. Clinically, there is a distinction between primary complaints of back pain, which are typically positional and mechanical, compared to neurologic and clinical signs of root entrapment and root compression with radiation to the buttock or leg [9]. The anatomic source of back pain can be muscular, from irritation and abnormalities of the disc or related to irritation of the facet articulations [9-10]. Over time, abnormal spinal biomechanics plays a large role in shifting the focus of disc pathology from the disc to the facet joints [11-12]. In the normal disc, the anterior column including the disc bears 92% of the axial load and the posterior facet only 8%, but with disc degeneration, the load shifts posteriorly, increasing the load on the facet joints five-fold to almost 40% so the facet joints become another source of back pain [10-13]. With degeneration of the lumbar disc, there is also loss of the disc's normal visco-elastic properties which allow expansion and contraction of the spinal segment with motion [12-14]. This loss of elasticity of the disc leads to segmental stiffness, and when combined with para-spinal muscle weakness alters the normal spinal mechanics and leads to subtle inflammatory changes in the disc annulus and the capsule of the lumbar facet joints that can cause back pain [14-15]. Any surgical procedure involving the disc and nucleus, no matter how minimal, frequently leads to loss of height of the disc. Experimentally, loss in disc height initially reduces tension on the posterior annulus, and possibly the adjacent compressed nerve root, but paradoxically over time can lead to foraminal narrowing and facet degeneration that can develop into more chronic nerve root compression and nerve entrapment [14].

Radiologic evaluation of the lumbar spine and disc

**Figure 1:**
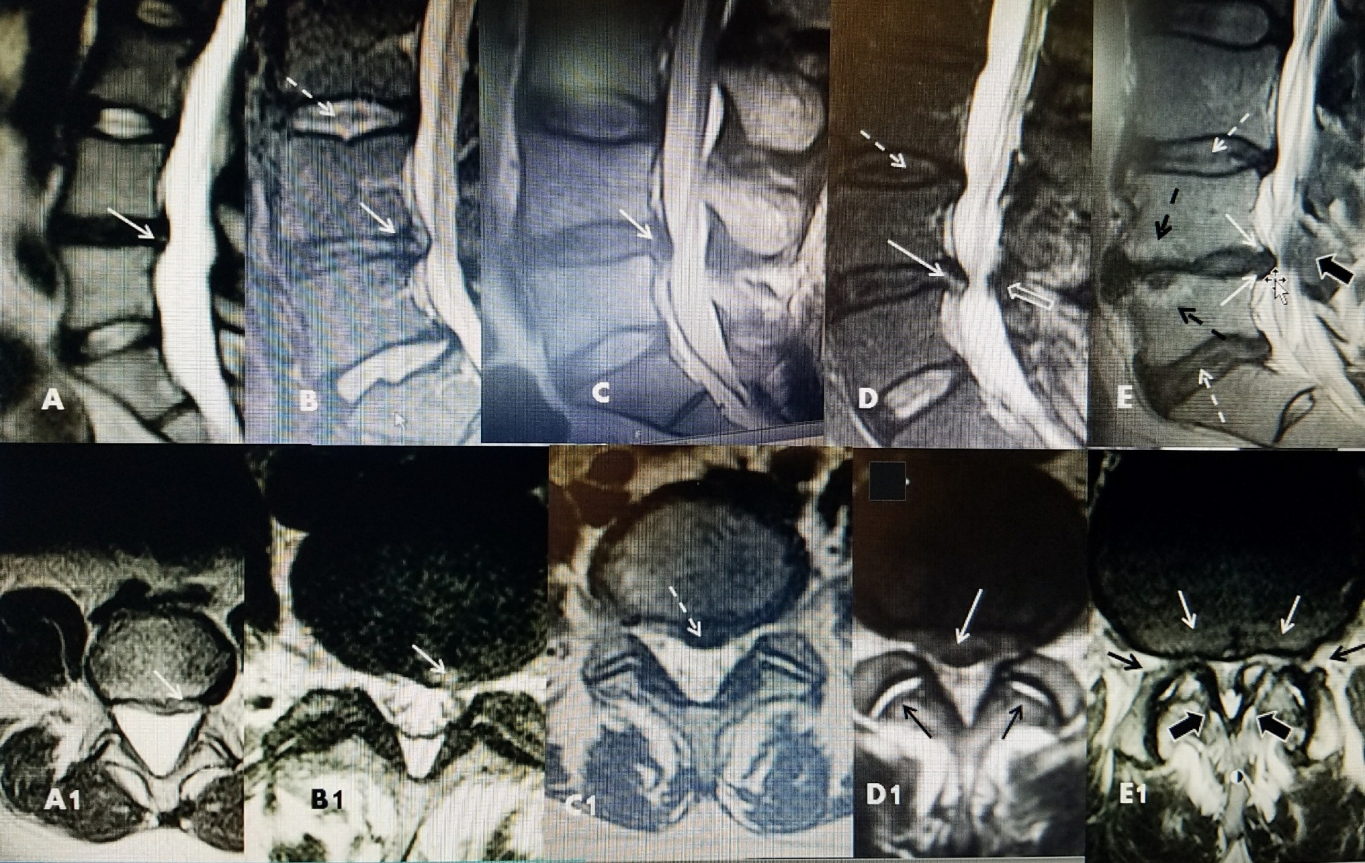
Range of sizes of L4-5 herniated discs A/A1: Sagittal and axial magnetic resonance image (MRI) of small L4-5 disc with 2 mm herniation but marked T2 signal change indicating disc dehydration and dessication. White arrow shows midline herniation. B/B1: Sagittal and axial MRI showing more distinct annular tear and sub-annular slightly superior herniation (solid white arrow). There is some slight narrowing of the vertical height of the disc space. There is a dessicated line in the center of the disc space above indicative of early Pfirrmann changes (dotted white arrow). C/C1: Sagittal and axial MRI showing midline 3-4 mm herniation with more distinct part inferiorly into the epidural space under the posterior longitudinal ligament. D/D1: Larger 6 mm herniation that is still contained but in the anterior epidural space (solid white arrow). There is minimal but distinct posterior ligamentum hypertrophy. The combination is causing localized canal stenosis (open white arrow). There is signal change in the disc above indicative of early dessication (dotted white arrow) with a small annular bulge. On the axial film there is high intensity fluid in both facet joints indicative of joint inflammation (black arrows). E/E1: MRI showing marked disc space narrowing at L4-5 with Modic changes in the anterior half of the both endplates (dotted black arrow). There is more distinct annular fibrotic thickening ( 2 solid white arrows) causing ventral compression (dotted cross and small white arrow) in the anterior epidural space. Posteriorly there is narrowing secondary to hypertrophied ligamentum. The facet joint is better seen on axial view in E1 (black arrow with white border) and there is a large calcified portion of the facet indenting the medial canal. The combination causes marked canal stenosis. On axial view there is bilateral neural foramina stenosis (solid black arrows).

There are numerous classifications and grading systems based on CT and MRI scans to ‘quantify’ the severity of disc changes and degeneration. This was refined into a nomenclature system by Phirrmann grading both disc height and hydration on MRI [9]. There is also the description and classification of Modic endplate changes seen with inflammation, fibrous, and fat ingrowth that is associated with disc degeneration on MRI [8]. Several groups have integrated both descriptions [9, 12]. The actual nomenclature used often adds to the confusion when reviewing treatments if reporting is not consistent [6]. Reliability of the MRI interpretation across observers is also an issue as shown in examples of grades of pathologic disc change at L4-5 level [2, 7] (Figure 1).

**Figure 2:**
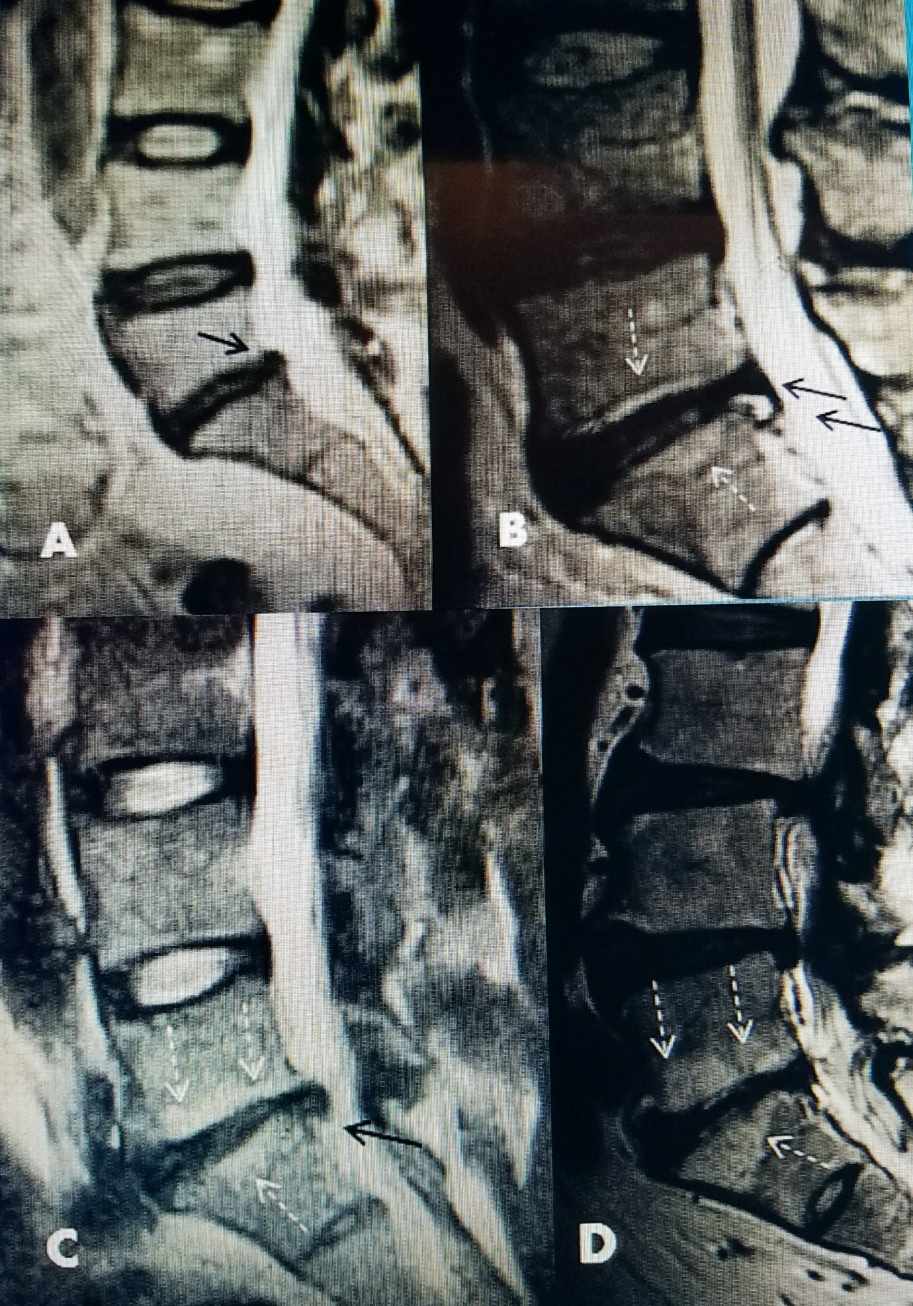
Examples of L5-S1 disc degeneration A: Sagittal T2 magnetic resonance imaging (MRI) showing small posterior herniation (black arrow) with some T2 signal change in both L5-S1 and L4-5 disc. B: Mild narrowing L5-S1 disc with small subligamentous herniation (solid black arrow) and bulging thickened annulus above (dotted arrow). There is very early minor endplate Modic change in middle of L5-S1 endplates (dotted white arrow). C: Progressive narrowing at L5-S1 with annular bulge rather than disc herniation (black arrow) and more marked Modic changes in endplate (dotted white arrows). D: More severe Modic change (dotted white arrows) with no evidence of disc or annular herniation. There is associated stenosis at L4-5 and L3-4 that is above the L5-S1 degenerative disc.

CT and MRI scanning demonstrate a range of pathology from internal disc disruption with dehydration changes on MRI, to simple annular disc bulge, associated with degrees of disc space narrowing, Modic endplate changes and facet hypertrophy that eventually leads to foraminal narrowing and entrapment of the nerve root [2, 4, 6]. Since it is possible to have small disc degenerations with severe symptoms and large herniations without symptoms, the mechanism that produces pain is not directly related to anatomic findings or radiologic 'compression' [2, 4, 7]. Disc 'disease' is a progressive and degenerative process so trying to precisely relate CT and MRI findings with specific symptoms can be problematic. There is not a direct relationship between clinical symptoms, pain, and radiologic findings. The radiologic findings indicate the levels of spinal pathology; however, multiple large studies of MRI scans in asymptomatic people consistently demonstrate significant degenerative disc changes at one or more levels including disc space narrowing, evidence of disc degeneration with intra-discal signal changes and endplate Modic changes, whose frequency steadily increases with age [7-9]. Even when initially studied, during their first episode of pain, many patients are found to have multilevel changes in the spine [5-6, 9]. In large MRI reviews, disc signal loss was seen in greater than 50% of people over age 40; however small annular protrusions and fissures were seen across all age groups but didn’t increase with age [2, 5]. Lumbar disc height loss and non-quantified disk 'bulge' was estimated to increase by 1-2% per year after age 30 [5]. It has been found that disk degeneration ranged in prevalence from 37% of 20-year-olds to 96% of 80-year-olds and facet degeneration also markedly increased with age [8, 10, 14-15] (Figure 2).

**Figure 3:**
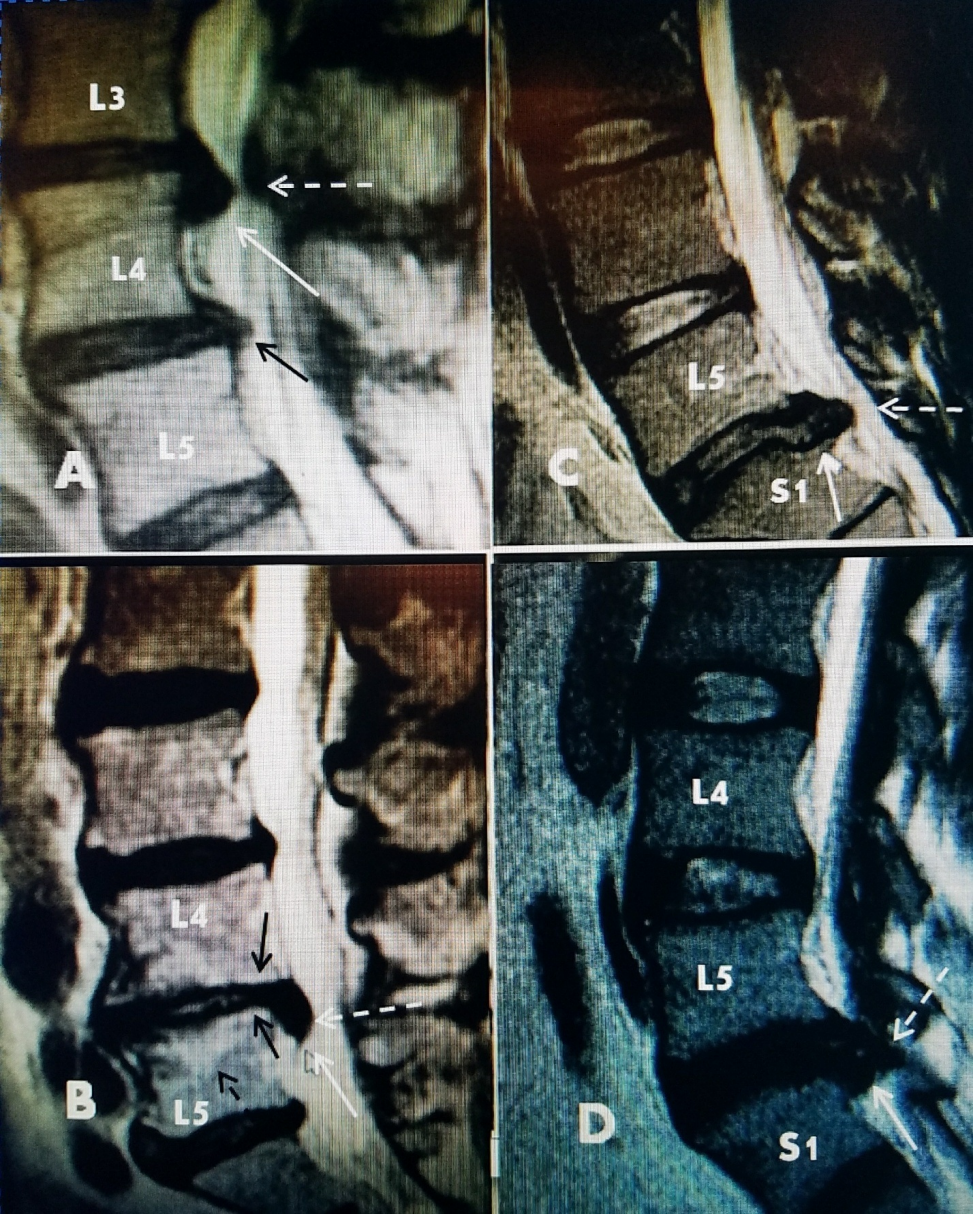
Examples of migrated and extruded disc fragments A: T2 sagittal magnetic resonance imaging (MRI) showing inferior extending L3-4 disc fragment (solid white arrow) and marked lateral spinal canal narrowing (dotted white arrow). There is also grade 1 spondylolisthesis at L4-5 with annular thickening (solid black arrow). B: Sagittal MRI showing inferior extending L4-5 disc fragment (solid white arrow) compressing and lifting the dura (dotted white arrow). There is associated L4-5 disc space narrowing (small solid black arrows) and early Modic endplate change especially at L5 (dotted short black arrow). C: T2 sagittal MRI showing large posteriorly extending L5-S1 disc (solid white arrow). The disc has marked T2 signal change and loss of fluid signal seen with disc dessication and fluid loss. The disc is extending directly posteriorly (dotted white arrow). D: MRI showing a large posterior lateral extrusion of L5-S1 disc (solid white arrow). The lateral recess is completely obstructed posteriorly adjacent to the facet joint (dotted white arrow).

Studies have compared the stages and relative size, location, and type of herniated discs to try and predict and guide the surgeon in selecting the type of surgery best suited for the particular patient. These studies attempting to predict outcomes of both conventional and microsurgical disc surgery based on disc size measured on MRI studies have produced inconsistent findings [16]. Location of the disc, disc space narrowing, the presence of larger eccentric disc fragments, the degree of narrowing or compromise of the axial spinal canal and associated bone changes, especially around the facet joints, are some of the findings examined to analyze how they affect outcomes [17-19] (Figure 3).

The size of the canal and relative canal compromise has been used to predict need for surgery, outcomes and estimate the clinical impact of radiologic findings [18]. A long-term eight-year MRI follow-up study showed that despite resolution of symptoms, frequently the post-procedure MRI did not change in as many as 65% of cases over 12 months and actually decreased in only 17.5% [16]. Preoperatively, finding more than 1/3 decrease in axial canal size was a better predictor of outcomes compared to only small herniations and Modic change with endoscopic or microdiscectomy lumbar surgery [16-17]. Large disc herniations and extruded or free fragments were clearly seen in cases with large decreases in canal dimensions [18-19]. However, there are numerous reports showing patients being completely asymptomatic even with a large disc herniation or severe multilevel changes on MRI scan, so a large radiologic defect by itself may not directly cause pain or symptomatic root compression. When symptomatic, large disc herniations or fragments frequently push the dura and nerve roots away from the foramina and anterior epidural space. Observation of the root during open or endoscopic surgery find that the root is swollen, inflamed with loss of normal cerebrospinal fluid pulsations all of which indicate root entrapment [19-21]. CT and MRI help to evaluate the position of migrated fragments or detect recurrent disc herniations which are critical in determining the best surgical approach, but often large disc fragments are found to have a remaining attachment to the annulus not seen on MRI scan [20-21]. Even after removal of large sequestered fragments either through open microsurgery or endoscopic surgery, post procedure MRI evaluations often reveal persistent radiologic defects even when the clinical symptoms have resolved [22]. Similarly, after automated percutaneous discectomy, there may remain radiologic annular bulging even as biomechanical studies show a reduction in disc pressure [23]. It appears that the mere existence of radiologic and anatomic defects or disc space narrowing may not always be the cause of symptoms unless there is resultant root inflammation and entrapment or associated abnormal biomechanics altering pressure within the disc, leading to stretching of the annular capsule or the nearby nerve root.

Biomechanics and pathophysiology of the abnormal disc and its relationship to possible surgical procedures

Experimental work on the progressive changes that occur with disc degeneration demonstrate a change in water content with initial expansion or swelling of the nucleus, displacement of the posterior annular wall, followed by annular tears and later disruption of these same annular fibers leading to laxity of the capsule after the disc swelling resolves, or residual sequestered sub-ligamentous disc fragments or disc extrusions [2-3]. The laxity and tears in the annulus are a result of the gradual loss of the polyglycans that provide elasticity to the normal disc [24]. Annular tensile strength is provided by collagen fibers which aid in torsion strength, but this tensile strength deteriorates, leading to tears and fissures with annular bulges. This sequentially results in shifting of the spinal load on to the posterior part of the spinal segment [2, 10-11]. As the disc deteriorates, accompanied by loss of osmotic nutrition to the disc, a fibro-proliferative process leading to replacement of endplate bone marrow with fibrous tissue and fat further decreases the ability of the disc to osmotically receive nutrients [20]. Pathologic studies show these fissures and tears occur more in the posterior part of the disc, indicating that herniations do not equally develop in all directions but are associated with some shifting of disc nucleus posteriorly [10, 24-25]. Radiologically this is associated with disc space narrowing, facet joint fluid and eventually Modic endplate changes indicating more chronic inflammation and degeneration [8-9]. Over time, with repetitive load, there is eventual annular tissue failure, which is indicated as darkening on T2 MRI signal showing loss of disc fluid, desiccation and dehydration and posterior bulging of the annular wall. The annular fibers separate and fragment as the normal elastic tissues are replaced by fibrotic strands allowing parts of the nucleus to 'migrate' into the tears of the annulus causing more localized herniations. These structures are also highly sensitive. The posterior annulus and endplates are innervated by recurrent sensory branches from the dorsal root and the basi-vertebral nerve within the vertebral body [11-12]. It has been shown that there is a marked angiogenic and neural ingrowth response to inflammation with development of excess fine sensory nerve ingrowth primarily into the posterior annulus. These fine neural fibers are also sensitive to breakdown of polyglycans, which can cause an intense inflammatory reaction. This may be a possible source of axial back pain and spasm in cases of acute but minimally degenerated and contained discs [10, 25-26]. Hypothetically, any procedures of the nucleus that remove these breakdown products may decrease annular and neural inflammation. If symptomatic, usually with mid-back or slightly lateralized para-spinal pain, symptoms from these minor annular fissures and accompanying bulges are resolved via use of anti-inflammatory medication and physical therapy, even though the laxity and annular tears may persist. A small percentage of patients develop persistent axial back pain. If the disc protrusion persists and continually stretches the annular fibers, there can also be some referred radicular pain since the annulus is richly innervated from recurrent sensory branches by the dorsal root ganglion.

Anatomic Studies and What They Indicate About Nerve Root Entrapment

Anatomic studies show the disc is not oval, but more kidney bean shaped; and the two thinnest areas of the annulus are the posterior-lateral corners, which is where herniations are most commonly seen [10]. In contrast to the distinct boundary between the disc nucleus and annulus shown in diagrams and suggested by CT and MRI scan, detailed anatomic and pathologic studies of the intervertebral disc show that there is not a clear absolute line between the polyglycans in the nucleus and the collagen fibers of the annulus, but rather a gradual merging of the two materials. This is important in understanding what part of the disc protrudes or herniates [11]. This annular/nuclear complex can continue protruding into the epidural space, first under the posterior longitudinal ligament, or migrating away from the disc space and moving either superiorly or inferiorly. If the tear in the annulus continues to progress, it creates a localized bulge outward into the epidural space. It is at these early stages where simple internal discectomy has been used with small systems to decrease annular bulging and pressure. Nuclear material, and especially breakdown products such as polyglycans, can also migrate through the larger fissures and tears in the annulus leading to more direct nerve root compression and inflammation. Most likely, root symptoms are a mixture of inflammation and compression of the root from the posteriorly bulging annulus explaining why some defects are painless, while others are painful, and not directly related to the absolute site of the radiologic defect. With disc degeneration and disc space narrowing, chronic changes in the ligaments and facet overgrowth lead to more foraminal compression. All these structures surround the exiting nerve root cause foraminal stenosis and radicular pain [14]. More extreme tears allow nuclear and annular material, as well as breakdown products of polyglycans, which are highly inflammatory, to pass through the posterior longitudinal ligament into the epidural space creating more inflammation of the nerve root with an 'attached' or free extruded fragment of disc [25]. More subacute and chronic inflammation leads to ingrowth of fine sensory branches from the recurrent sensory branch of the dorsal root and the basi-vertebral nerve that innervate the posterior spinal ligament and the endplates [15,25-27]. Relating these findings to clinical symptoms, in the earliest stage, patients may have annular inflammation and swelling with elevated intra-discal pressure leading to symptoms primarily of axial back pain. However, as the disc degenerates, and if the load shifts more posteriorly, then the synovium of the facet joints is affected, leading to facet inflammation, increased joint fluid, and progressive facet pain. At this early stage, if there is no evidence of neurologic root compression, using nonsteroidal anti-inflammatory (NSAID) and gabapentin to reduce neural inflammation and possibly image-guided steroid injections near the inflamed root, can be effective regardless of the actual radiologic size of the disc herniation [2-3, 15]. It is plausible, that early treatment of capsular and root inflammation reduces the chance of progression to chronic fibrotic change [19]. The role of intradiscal biologics in treatment is still uncertain, but It is most likely at this early stage of disc degeneration where intradiscal or intra-annular biologics will be effective with or without disc decompression [3]. Long-term follow-up radiologic studies with MRI scans on conservatively treated patients have shown that paradoxically, many asymptomatic patients may still have large herniated discs or free fragments; and in follow-up radiologic studies, many disc defects slowly dehydrate, shrink and retract [19, 24-25]. The importance of understanding the underlying pathologic process and the natural steps of progression of disc degeneration is the basis for the pursuit of minimal disruptive procedures and efforts to develop biologic and cellular regenerative treatments for the earliest stages of disc degeneration in hopes of arresting or even reversing this process [3, 28-29]. 

The discectomy systems

The natural history and evolution of disc herniation is generally in favor of symptomatic resolution; however, the degenerative process may continue while the patient remains clinically asymptomatic. This is supported by many radiologic studies showing increased frequency and levels of progressive radiologic change with age [4-5, 8-9]. Conservative measures with anti-inflammatory medication, exercise, and physical therapy should be the first and simplest level of treatment. If conservative treatment fails, following the evolution explained earlier, minimally invasive procedures within the disc or release of the anatomic compression of the nerve root within the spinal canal or neural foramen should be the target of surgery on the lumbar disc, whether through conventional open microsurgery or through posterior or transforaminal tubular minimally invasive procedures. In the more than 30 years that different systems have been available for removing a herniated lumbar disc through a posterior-lateral tubular system, there has not been an in-depth evaluation of effectiveness based on characteristics of the disc seen on MRI scan rather than a particular system [30-31]. Understanding that narrowing of the foramina, due either to narrowing and subsidence of the disc space or overgrowth of the facet joints or both, is a major factor in root impingement, decompression of the disc and especially the more ventral part of the superior facet and opening of the posterior foramina has become a key part of minimally invasive transforaminal procedures [30-32]. As a result, many of the more advanced endoscopic percutaneous systems now include a means for foraminal opening with progressively larger drills in addition to disc decompression and disc fragment resection. As in open surgery, root entrapment is associated with swelling and inflammation and signs of successful release, and freeing of the nerve root are demonstrated by the return of dural pulsation [21-22, 31-32]. Symptomatic relief is not always directly correlated with immediate resolution of the radiologic defect [2, 23, 27]. Post-procedure MRIs, especially in the first three to six months after surgery, often show residual radiologic defects [28]. Studies indicate that symptomatic relief and clinical improvement may initially be a result of a decrease in root inflammation after decompression of the nerve root [29]. This indicates that, as in open surgery, the focus on lumbar disc herniation surgery should be primarily targeting the actual release of the nerve root rather than only the removal of the central part of the disc.

Characterization of the Endoscopic and Tubular Systems

**Figure 4:**
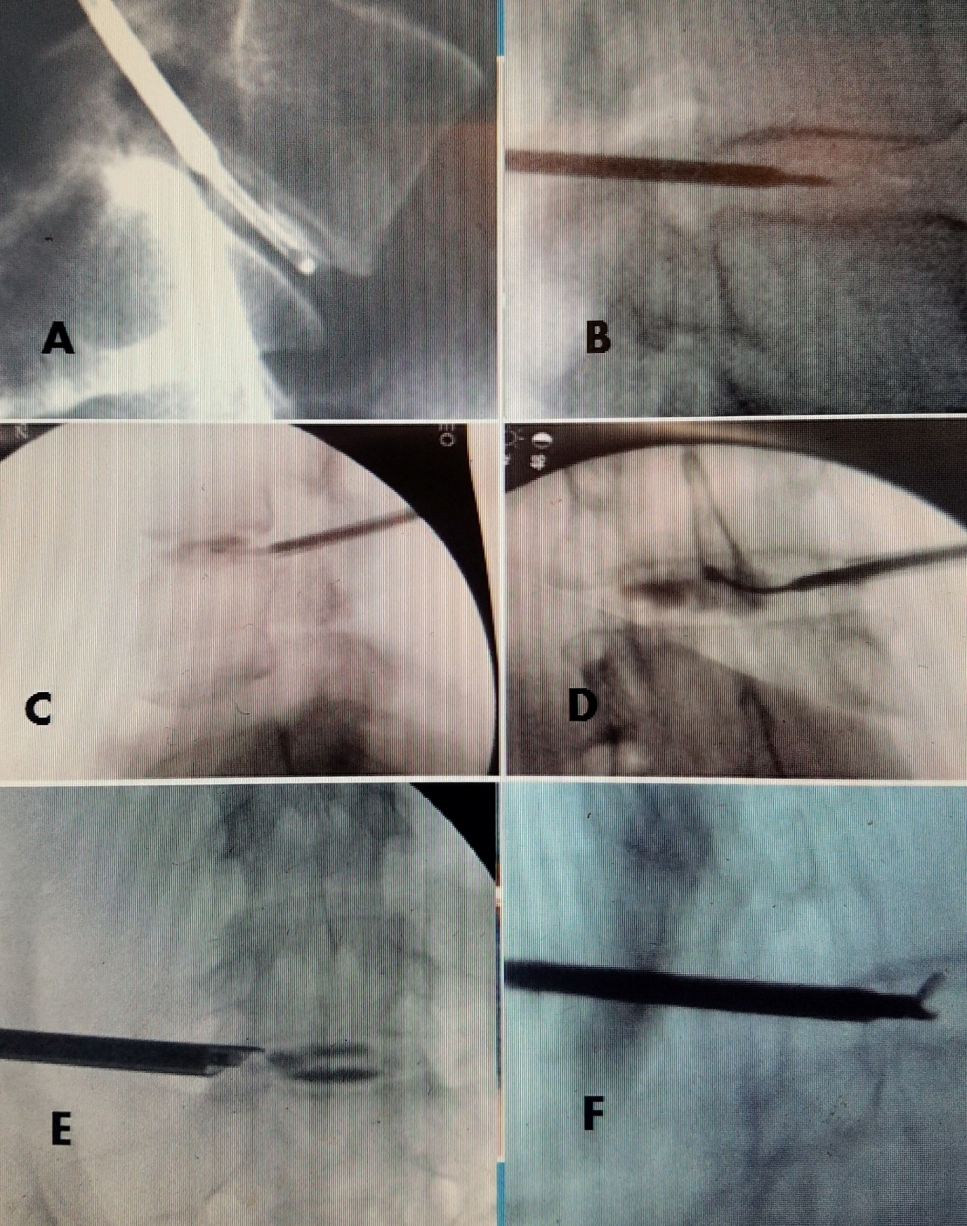
Intra-operative images of different endoscopic and foraminal tubular systems A: 2.1 mm aspirating cutter in the L5-S1 disc space for a contained disc. Note the probe takes up approximately 25% of the disc height and with movement can cover the entire vertical area of the nucleus. B: 2.4 mm straight cannula over guide wire into the L4-5 contained herniated disc. Note the size of the cannula relative to the disc height. C: 1.9 mm cannula placed after a discogram to identify nucleus under fluoroscopy. D: Anterior-posterior view of cannula with intra-discal curved curette crossing the midline within the nucleus. The nucleus is identified and localized with radiographic contrast material in a discogram. E: 6.0 mm lateral beveled transforaminal cannula at margin of L5-S1 after a discogram. Note the dimension of the cannula encompasses most of the vertical height of the disc space allowing easy movement of instruments through the cannula and within the disc. F: L3-4 disc using a 4.8 mm cannula with pituitary ronguer in posterior margin of disc space. The pituitary ronguer is able to move around the disc space making contact with both the superior and inferior extent of the endplates and remove more peripheral disc material.

All tubular minimally invasive systems access the disc through the inferior posterior lateral corner of the intervertebral foraminal through what is named ‘Kambin’s Triangle’ [32-33]. Guide wires and fluoroscopic image guidance alone or combined with visualization with endoscopes is used to work directly in and through the intervertebral foramina then advancing into the posterior-lateral corner of the disc annulus to enter the disc itself or the lateral and central epidural space to approach larger disc herniations and fragments [32-33]. There are two basic groups of systems: the first group consists of systems that use small tubes between 1.5 to 2.5 mm that macerate and remove soft disc by aspiration, or combined with minimal disc removal with a fine pituitary rongeur and possibly adding radiofrequency capsule ablation [1]. When using the larger, endoscopic systems, with proper positioning, the surgeon is able to use a series of progressively larger fine drills to enlarge the neural foramina by removing part of the superior facet. This enables the surgeon to insert up to an 8 mm cannula into the posterior-lateral epidural space and then along the central margin of the disc space [31, 33]. Guided by radiologic studies with MRI and CT, the surgeon knows if the herniated part of the disc is contained and in direct continuity with the nucleus, if the size of the proposed tube adequately allows the surgeon to cover the central and peripheral areas where the disc or fragment is located and if the surgeon is able to visualize and manipulate throughout the entire disc space. Larger or displaced herniated discs require these larger systems allowing insertion of graspers and micro-curettes. The instruments can also enter the intervertebral disc space [34]. Larger discs commonly require the use of pituitary rongeurs and curettes to remove disc material in the neural foramina or the spinal canal and results are comparable to open micro-laminectomy and discectomy [35-38]. The foraminal and facet drills make widening the foramina possible and help decompression of the lateral nerve root. Visualization using endoscopic systems combined with fluoroscopy guidance allows removal of large migrated and extruded disc fragments (Figure 4).

What amount of abnormal disc nucleus material is necessary to be removed for the best outcome

**Figure 5:**
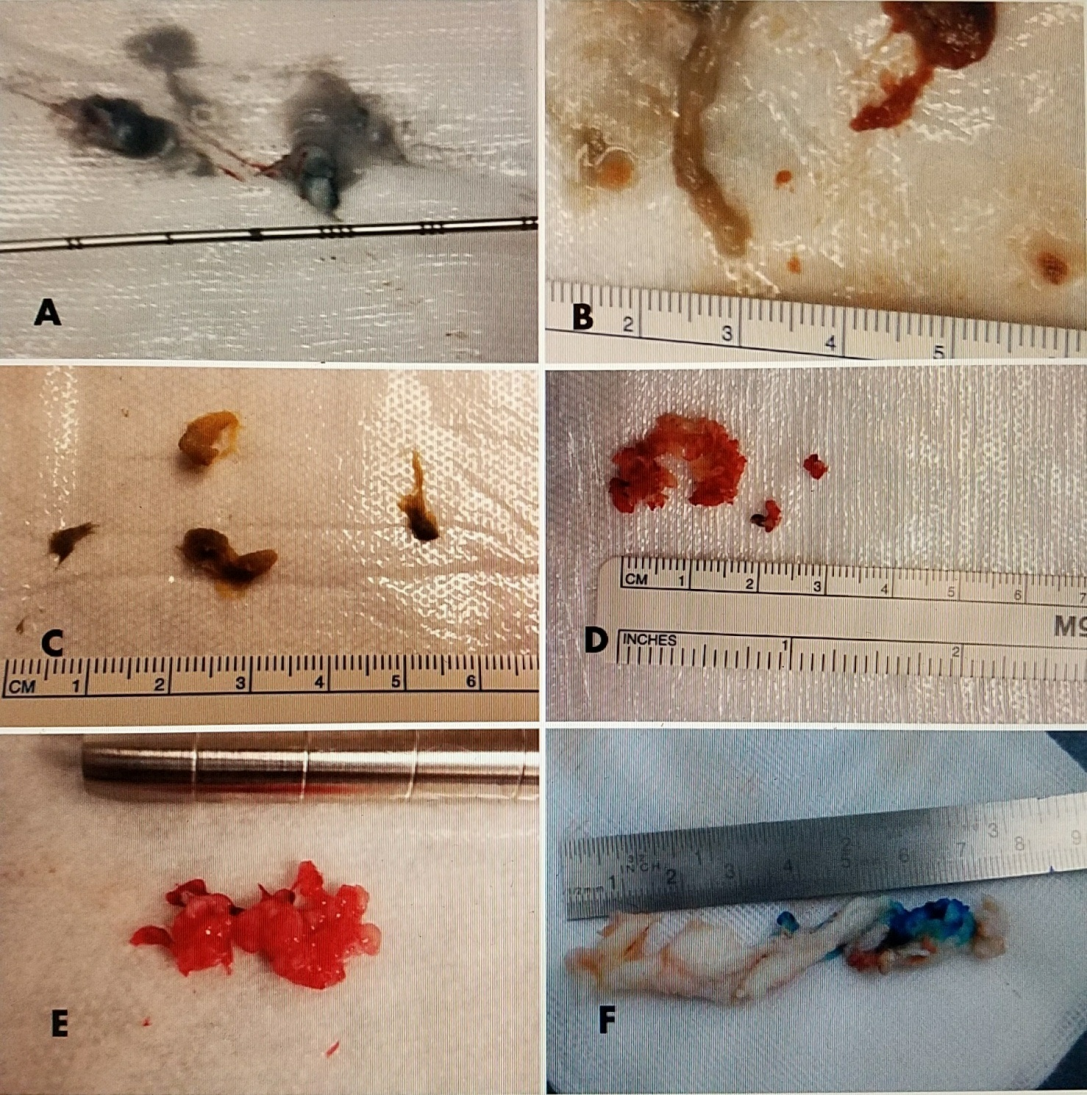
Surgical specimens obtained with various size tubular disc excisions All measurements are based on millimeter and centimeter marker. A: Macerated nuclear material aspirated with a 1.6 mm probe. B: Aspiration discectomy combined with use of 2.1 mm micro-pituitary forceps. C: Disc material obtained using small 2.1 mm pituitary forceps in a 2.4 mm cannula. D: Disc fragments obtained using a 3.4 mm cannula using 2.8 mm pituitary forceps. E: Disc fragments obtained using a 4.2 mm cannula with 3.5 mm pituitary forceps. F: Extruded free fragment stained with Indigo Carmen extracted thru 6.6 mm cannula and endoscopic system.

The ultimate question is what and how much nuclear material must be removed to get both short-term and long-term improvement in the patient's symptoms. This is also determined by understanding if the main pathology is disc enlargement and annular swelling or root compression from displaced disc and bone. Using a direct visualization endoscopic system, it is possible to visualize the nerve root and make sure it is released from capsular adhesions and fibrosis. After decompression of the root, there should be the return of free pulsations indicating there is no further pressure and root entrapment. In cases with larger disc herniations and fragments, as in open microsurgery, the key step is identifying and visualizing the entrapped nerve root, making sure it is free of fibrous adhesions and any remaining compressing disc fragments, or bony constriction from facet overgrowth [31-34]. Specimens obtained can vary depending on the type of system used (Figure 5).

**Figure 6:**
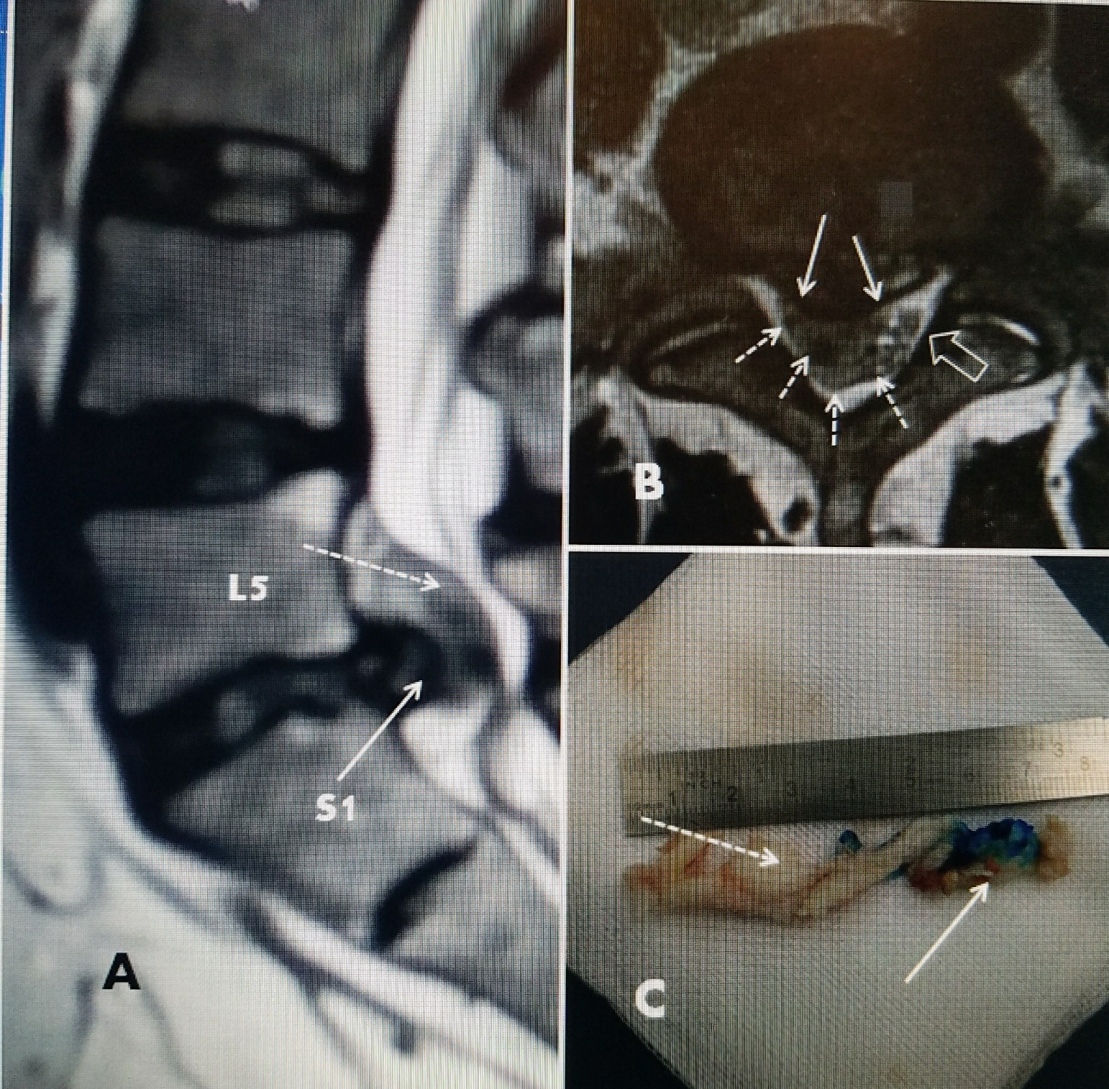
Large partially extruded L5-S1 disc with surgical speciment A: Sagittal T2 magnetic resonance imaging (MRI) showing the large posterior L5-S1 disc herniation (solid white arrow) surrounded by a lighter shadow of the more superior placed extruded fragment (dotted white arrow). The dural sac is pushed lateral and posterior. B: Axial MRI scan showing the posterior and lateral disc (solid white arrow) with surrounding extruded piece of disc is more posteriorly and pushing the dural sac completely to the opposite side (open white arrow). C: Surgical specimen removed through the endoscope under local anesthesia. Indigo Carmen, blue dye, had been injected into the contained part of the disc and can be clearly seen (solid white arrow). The extruded but still attached fragment that was in the spinal canal can be seen (dotted white arrow). The annulus of the disc and attached fragment is over 7 cm in length.

Very large disc herniations and even recurrent disc herniations even that have a fragment that has migrated or moved away from the disc space can be approached through tubular endoscopic systems and disc removal. Although it is possible to approach the more posterior and medial part of the disc with an endoscope, the para-median and lateral disc are ideally suited for this approach [36-38]. In these cases, the large disc fragment pushes the dura posteriorly and away from the herniated side so when combined with foraminoplasty, there is endoscopic access to the lateral recess and the ventral floor of the neural foramina [37]. This allows a direct approach to the fragment. The exiting nerve root can be identified going superiorly and posterior. The inferiorly passing nerve is more medial and descending to the next spinal level. The exiting root is not affected unless there is a direct posterior-lateral and superior herniation. In this case, the nerve root is pushed up and lateral (Figure 6).

Using both endoscopic visualization and simultaneous fluoroscopic imaging enables the surgeon to know whether the cannula is properly aligned towards the fragment in the proper direction indicated by MRI. This allows the experienced endoscopic surgeon to approach and remove large and extremely displaced ventral epidural disc fragments, perform a partial facetectomy to free the nerve root within the neural foramina, and remove recurrent discs or place interbody graft material for intervertebral fusion [34-38]. Even in cases with recurrent disc herniations, endoscopic surgery can be successful [20-21, 24, 35, 37, 39]. By manipulation of the angle of approach to the disc space, the cannula can be directed toward the disc fragment that has recurred even if it has migrated out of alignment with the disc space [33, 37]. More studies are needed to show at what stage is the threshold of change from local inflammation to fibrosis with the degeneration of the lumbar disc. The development of fibrosis and its effect on the nerve root followed by more chronic changes, leading to the development of bone and facet overgrowth associated with narrowing of the intervertebral foramina, shifts the goal of transforminal procedures to nerve root decompression and relieving root entrapment rather than simple discectomy. This has the largest impact on the decision of which type of procedure is best suited for a particular patient. As reviewed in this article, understanding the progressive mechanical and pathologic changes that occur with disc degeneration, even without symptoms, is an important step. The development of angiogenic and neural proliferative reactions in response to tissue inflammation from the breakdown of nuclear disc substances may be where biologics will be effective in treating the abnormal disc at its earliest stages not only to treat 'pain' but to have an effect on the degenerative process {3, 25-27]. It is possible biologics will be combined with these minimally invasive approaches to arrest or reverse both disc degeneration as well as the localized changes that result from the inflammatory process around the disc capsule, annulus, and nerve root.

## Conclusions

Rather than concentrating on the specific tubular instrument system used, the underlying pathology seen on CT and MRI as well as understanding how it relates to the process of disc degeneration and the patient's clinical symptoms should be the focus of the decision regarding the best type of percutaneous transforaminal system to be selected. The more experience the surgeon has with using different systems, understanding their capabilities and limitations of the instruments and how it meshes with the goal of the surgery, allows the maximum use of these minimal surgical approaches and techniques. Over the 30-plus years that tubular and endoscopic systems have evolved, it is clear that many different types of smaller herniated and partially degenerated discs can be effectively and safely de-bulked or removed using these minimally invasive procedures. As these procedures keep evolving, removal of large or migrated disc fragments is more routine. New systems enable the surgeon to do a lateral partial foraminotomy and the possibility to perform interbody fusion. The major advantage of these procedures is complete sparing of damage to the posterior supporting muscles and ligaments, the ability to perform them under local anesthesia with minimal sedation, and in an ambulatory setting. All these elements lead to a faster recovery and preservation of spinal stability. However, the surgeon must also integrate planning the type and severity of the patient's symptoms using the available diagnostic information to identify the specific level and degree of segmental spinal pathology that is creating the patient's problem. It is critical to take into consideration the degree of associated disc space narrowing and facet hypertrophy to guarantee the nerve root is adequately decompressed. The endoscopic surgeon must be able to accomplish this, just as in open surgery, but using minimally-invasive endoscopic procedures that are targeting the disc, disc fragments, and the intervertebral foramina. Proper visualization and release of the entrapped nerve root is a critical step in these procedures. 
